# Diagnosis of cervical plexus tumours by high-frequency ultrasonography

**DOI:** 10.1186/s12880-021-00682-5

**Published:** 2021-10-14

**Authors:** Wenqing Gong, Jing Wang, Liwei Huang, Xu Yang, Dingzhang Chen, Minjuan Zheng

**Affiliations:** 1 Department of Ultrasound, Xijing Hospital, Fourth Military Medical University, No. 127 Changle West Road, Xi’an, 710032 Shaanxi China; 2 Department of Special Clinic, Rehabilitation Center, Joint Logistics Support Force of PLA, Lintong, 710600 Shaanxi China; 3 Department of Radiology, Xijing Hospital, Fourth Military Medical University, Xi’an, 710032 Shaanxi China

**Keywords:** Cervical plexus, Tumour, High-frequency ultrasonography

## Abstract

**Background:**

Cervical plexus (CP) tumours are difficult to diagnose because of atypical symptoms. This study aimed to summarize the features of a normal CP and CP tumours observed on high-frequency ultrasonography.

**Methods:**

The ultrasound data of 11 CP tumour patients and 22 normal volunteers were collected. All 11 patients underwent magnetic resonance imaging (MRI), and 4 patients also underwent computed tomography (CT). The imaging data were compared with surgery and pathology data.

**Results:**

The C7 vertebra and bifurcation of the carotid common artery (CCA) were useful anatomic markers for identifying the CP. In contrast to the C1 nerve (22.7%), the C2-4 nerves were well displayed and thinner than the brachial plexus (*P* < 0.05). CP tumours were more common in females (72.7%) and generally located at C4 (72.7%) on the right side (81.8%). Additionally, the nerve trunk in tumour patients was obviously wider than that in normal controls (7.49 ± 1.03 mm vs 2.67 ± 0.36 mm, *P* < 0.01). Compared with pathology, the diagnostic rates of CP tumours by MRI, CT and high-frequency ultrasound were 72.7% (8/11), 25% (1/4) and 90.9% (10/11), respectively.

**Conclusions:**

The diagnosis of CP neuropathy is accurate and reliable by high-frequency ultrasound, and the C7 vertebra and bifurcation of the CCA are useful anatomic markers in CP ultrasonography.

## Background

The cervical plexus (CP) is located in the neck beneath the sternocleidomastoid muscle and comprises a coalition of nerves originating from C1 through C4 [1, 2]. Because of the higher location and atypical symptoms, most patients with CP tumor went to clinic for neck mass, some with concomitant pain, and CP masses are quite similar to lymph node or thyroid nodules upon palpation, so the presurgical diagnosis of these tumours is challenging [3]. The diagnosis of CP tumours relies on the clinical history, physical examination, electromyogram and diagnostic imaging [4]. Electromyogram was lack of specific manifestation, magnetic resonance imaging (MRI) offers high-resolution visualization of CP tumours; however, the availability of MR neurography may be limited, and it is costly. Therefore, the clinical diagnosis of CP tumours remains difficult.

As a valuable technology, high-frequency ultrasonography provides superior imaging, with resolution up to 30 µm, which is sufficient for establishing the precise type and level of nerve injury [5]. We successfully used high-frequency ultrasound to diagnose brachial plexus (BP) closed injuries and neoplasms [6]. Previous studies on the ultrasonic imaging of a normal CP or the diagnosis of CP tumours are rare, with the exception of some case reports [7], and CP tumours are often misdiagnosed as lymph nodes [3, 8]. Thus, this study aimed to summarize the ultrasound characteristics of a normal CP and CP tumours to provide references for CP tumour diagnosis.

## Methods

### Study subjects

From January 2017 to December 2019, eleven patients with CP masses (8 females (72.7%) and 3 males) who were diagnosed with CP tumours were enrolled. The patients were referred to the clinic for a palpable mass, arm numbness or pressing pain. All patients underwent high-resolution ultrasound neurography and MRI. Fourth patients also accepted Computed Tomography (CT) scanning. The average patient age was 40.73 ± 13.24 years (15–54 years). All 11 participants were confirmed to have CP tumours by surgery and pathology. Another 22 healthy adult volunteers (aged from 17 to 49, 37.55 ± 13.92 years) were recruited to assess measurements of a normal cervical nerve.

### Equipment and methods

MRI examinations were performed with the patient in the supine position with the head in a neutral position using a MAGNETOM Avanto 1.5 T MRI system (Siemens Healthcare, Erlangen, Germany). Sagittal T1WI, sagittal T2WI and axial T2WI for cervical spine were used as standard protocol. In addition, short inversion time inversion recovery sequences to suppress fat in paraspinal soft tissue, FLASH, and T1W FAT-SAT sequences were also included wherever necessary. 4 patients had taken a spiral CT scan (Somatom Definition Flash, Siemens Healthcare, Forchheim, Germany) from the top of the skull to the superior border of the aortic arch were reviewed. Images were reconstructed with a slice thickness of 1 mm and an increment of 0.8 mm. All images were analyzed by experienced radiologist (Xu Yang, Liwei Huang).

GE Logiq 9 ultrasound system with a 6–12 MHz linear array transducer (GE Medical Systems, Milwaukee, WI, USA). Ultrasonography was performed by two experienced sonographers (Jing Wang, Dingzhang Chen). The subjects were examined on both sides of the neck in the supine position. The CP nerve was fully scanned at the levels of the intervertebral foramen, roots, trunks, and visible terminal branches in the longitudinal and transverse planes. The diameters of the different levels of the CP nerve were measured in all subjects (healthy volunteers and those with CP tumours), and the size, echogenicity, Doppler blood flow and connection to the nerve of the masses were also evaluated.

Adler grading [9, 10] was used to classify Doppler blood flow in the tumour as follows: Adler 0 refers to that no obvious blood flow signal; Adler I refers to 1 or 2 small blood vessels with a diameter of 1 mm are detected; Adler II refers to that 3 or 4 small blood vessels are detected; Adler III refers to that more than 4 blood vessels, or the blood vessels are intertwined into a network are detected.

### Statistical analysis

Statistical analyses were performed using statistical software (SPSS for Windows, version 21.0; SPSS, Chicago, IL, USA). Continuous variables are expressed as the mean ± standard deviation (SD), and categorical variables are expressed as percentages. The variables were compared using the *t* test or Fisher’s exact test: *t* test was used to compare diameter of normal CP and BP nerve, and Fisher's exact test was used to compare difference between mass location and colour Doppler grade. Probability (*P*) values < 0.05 were considered significant.

## Results

### Ultrasonography features of a normal CP nerve

**Fig. 1:**
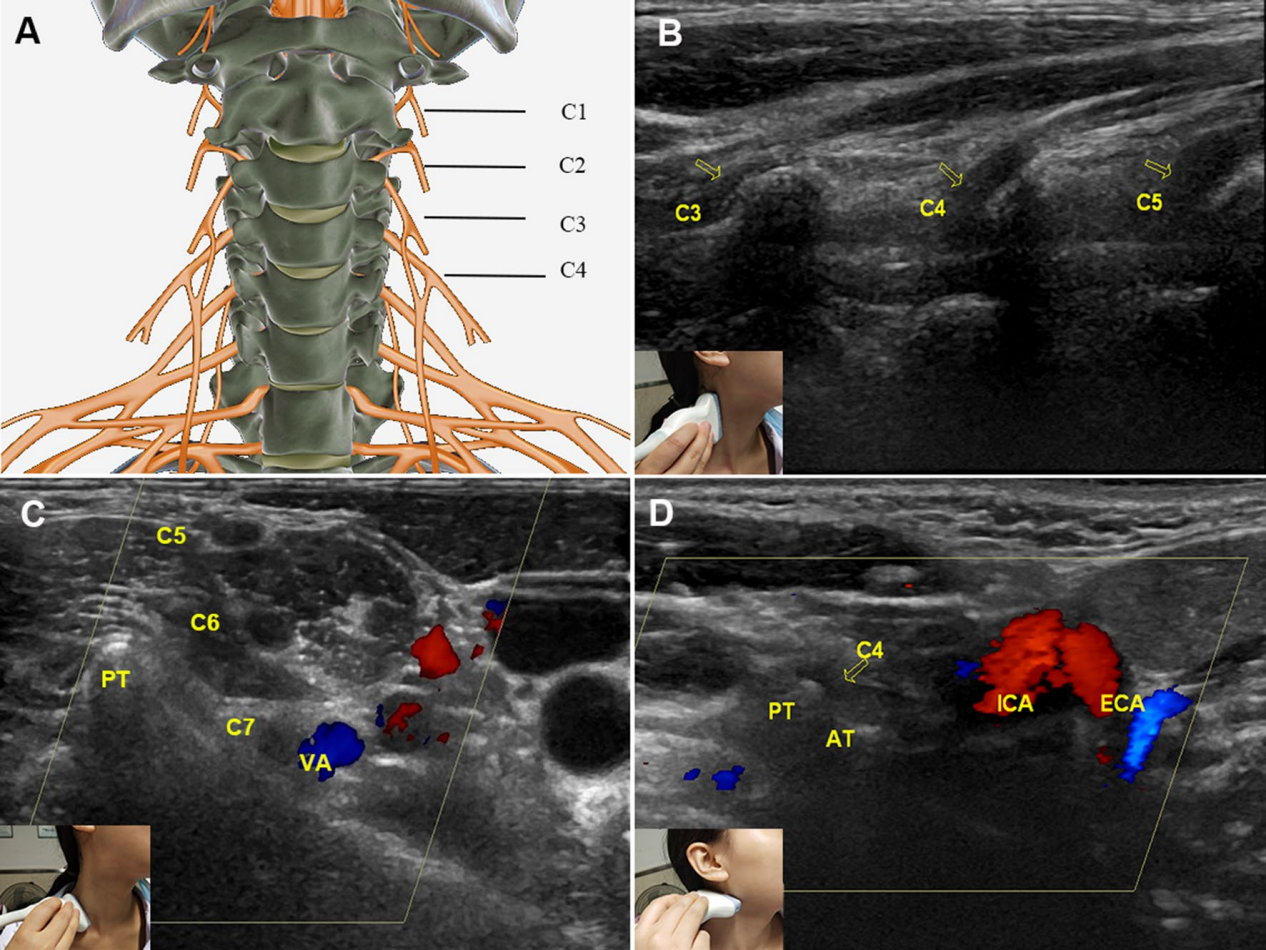
Normal cervical plexus and its anatomical markers. **A** Anatomic diagram of the CP. **B** Longitudinal view of the C3–C5 roots (arrows), which appear as tubular hypoechoic structures with echogenic walls and a fibrillar texture. **C** The C7 vertebra was used as an anatomical marker to identify the CP. Arrows point to cervical nerve C7 (transverse view, arrow) and the PT. **D** The bifurcation of the common carotid artery served as another anatomical marker for identifying the CP (C4 level); the arrow indicates C4. *PT* posterior tubercle, *AT* anterior tubercle, *VA* vertebral artery, *ECA* external carotid artery, *ICA* internal carotid artery

The ultrasound display rates of C2, C3 and C4 were 100%, but that of C1 was 22.7% (5/22) due to its deep location (Fig. 1A, B). We found that two land markers were useful in identifying the CP. (1) The C7 vertebra: C7 is unique because it has a posterior tubercle only and can easily be found (Fig. 1C); then, C4-1 (above) can be confirmed from bottom to top. (2) Bifurcation of the common carotid artery (CCA): The bifurcation of the CCA is at the C4 level, which can be used as a land marker for C4 recognition (Fig. 1D). Normal CPs appeared on transverse views as round or elliptical hypoechoic structures with a hyperechoic rim. Viewed longitudinally, they appeared as tubular structures with a hypoechoic background (Fig. 1B). The diameter of the CP was less than that of the brachial plexus (Table 1).

**Table 1:** Measurements of normal cervical nerve roots and nerves by ultrasonography (n = 44, 22 subjects, bilateral)

Cervical nerve	Trunk (mm)	Root (mm)	Intervertebral foramen level (mm)
Cervical plexus			
C2	2.46 ± 0.31	5.03 ± 0.70	7.65 ± 1.36
C3	2.75 ± 0.33	5.86 ± 0.82	9.21 ± 1.54
C4	2.83 ± 0.39	6.62 ± 1.13	11.18 ± 7.44
Mean	2.68 ± 0.38*	5.84 ± 1.04*	9.35 ± 4.72*
Brachial plexus			
C5	3.09 ± 0.47	7.33 ± 1.22	10.97 ± 2.55
C6	3.48 ± 0.39	7.54 ± 1.72	11.21 ± 2.90
C7	3.82 ± 0.55	7.57 ± 1.09	10.60 ± 2.74
C8	3.79 ± 0.61	7.24 ± 1.10	10.40 ± 2.58
Mean	3.55 ± 0.59	7.42 ± 1.31	10.79 ± 2.68

^*^P < 0.05, compared with the mean value of the brachial plexus

### Ultrasonography features of CP tumours

The clinical and ultrasound data of the 11 patients are shown in Table 2. The CP tumours presented as a single mass, and mainly located at C4 (72.7%, 8/11, *P* = 0.037) on the right side (81.8%, 9/11). Most tumours originated from the root level (63.6%, 7/11), with a mean diameter of 4.83 ± 1.62 cm. The diameter of the connected root nerve was markedly increased compared with that of the corresponding nerve on the healthy side because of oedema (7.49 ± 1.03 mm vs 2.67 ± 0.36 mm, *P* < 0.01). The intervertebral foramen was obviously enlarged.

**Table 2:** Clinical and ultrasound features of cervical plexus tumours (n = 11)

	n%, mean ± SD
Mean age (years)	40.73 ± 13.24
Female	72.7% (8)
Clinical manifestation	
Mass	54.5% (6)
Arm numbness	27.3% (3)
Pressing pain	18.2% (2)
High-frequency ultrasound	
Right side	81.8% (9)
Location of mass	
C4	72.7% (8)*
C3	27.3% (3)
Diameter of mass (cm)	4.83 ± 1.62
Diameter of nerve trunk of the tumour (mm)	7.49 ± 1.03
Intervertebral foramen level (mm)	12.70 ± 1.90
Clear boundary with envelope integrity	100% (11)
Colour Doppler grade	
I	9.1% (1)*
II	54.5% (6)*
III	36.4% (4)

^*^P < 0.05, compared with grade and with the C3 location

**Fig. 2:**
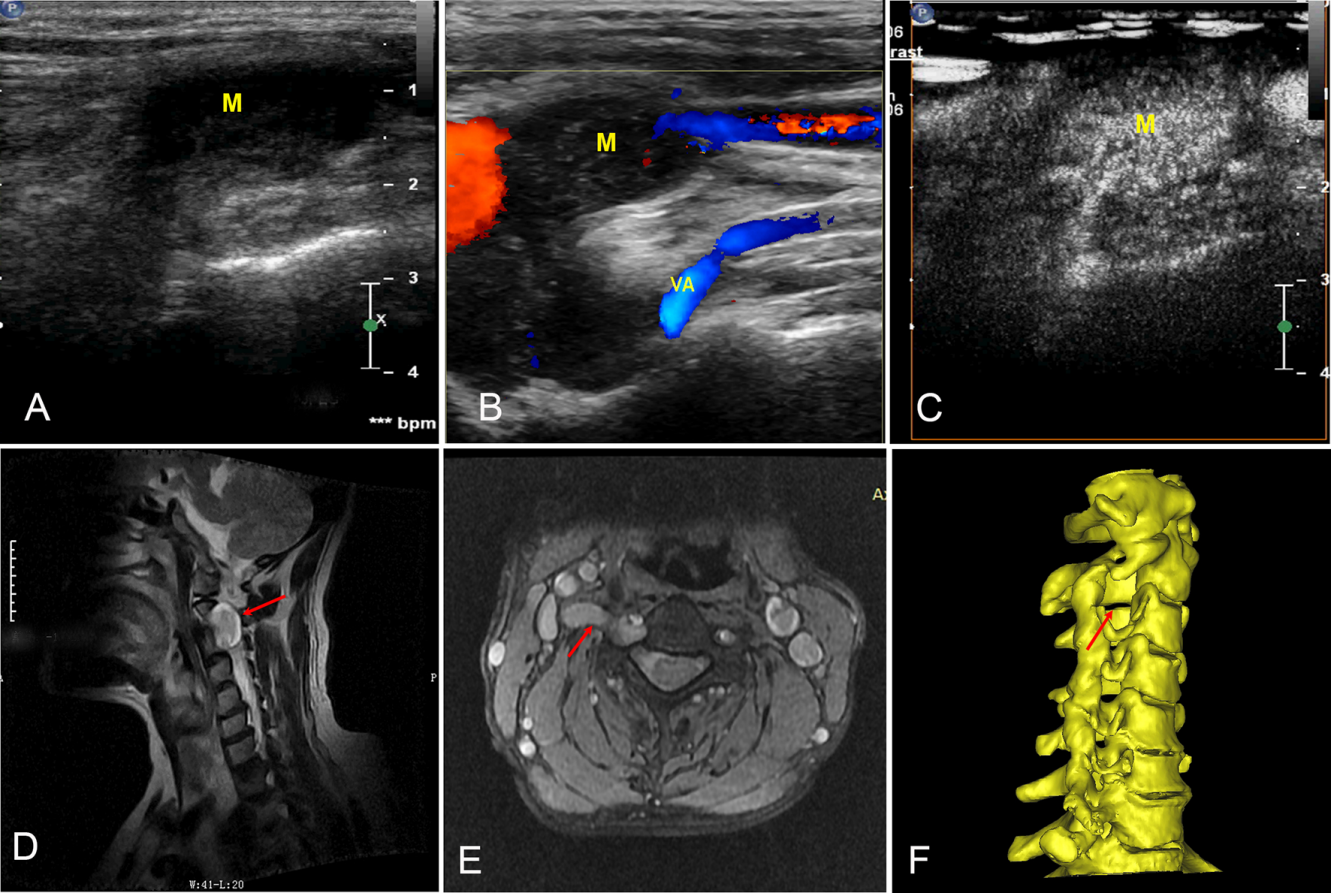
MRI, CT and contrast ultrasound images of cervical plexus schwannomas (C3 level). **A** 2D ultrasonography of the transverse process of the vertebra, where M is the neuroma lesion originating from the intervertebral foramen. **B** Colour Doppler ultrasonography showing the blood flow signal in the mass. **C** Contrast-enhanced ultrasound showing rich blood perfusion. **D**, **E** MRI sagittal and cross-sections of a CP mass: the arrow indicates the lesion growing outwards from the intervertebral foramen. **F** The arrow indicates the enlarged intervertebral foramen (3D CT reconstruction)

In high-resolution ultrasound imaging, most tumours were homogeneously hypoechoic (10, 90.9%) with a clear boundary and envelope integrity (11, 100%), and only one mass demonstrated an anechoic liquefaction zone. According to colour Doppler ultrasonography, moderate or strong blood signals were acquired from most tumours (grades II and III accounted for 90.9% of the total, and grade II alone accounted for 54.5%). Two patients who underwent contrast-enhanced ultrasound showed abundant blood perfusion (Fig. 2A–C).

### MRI, CT and pathology

**Fig. 3:**
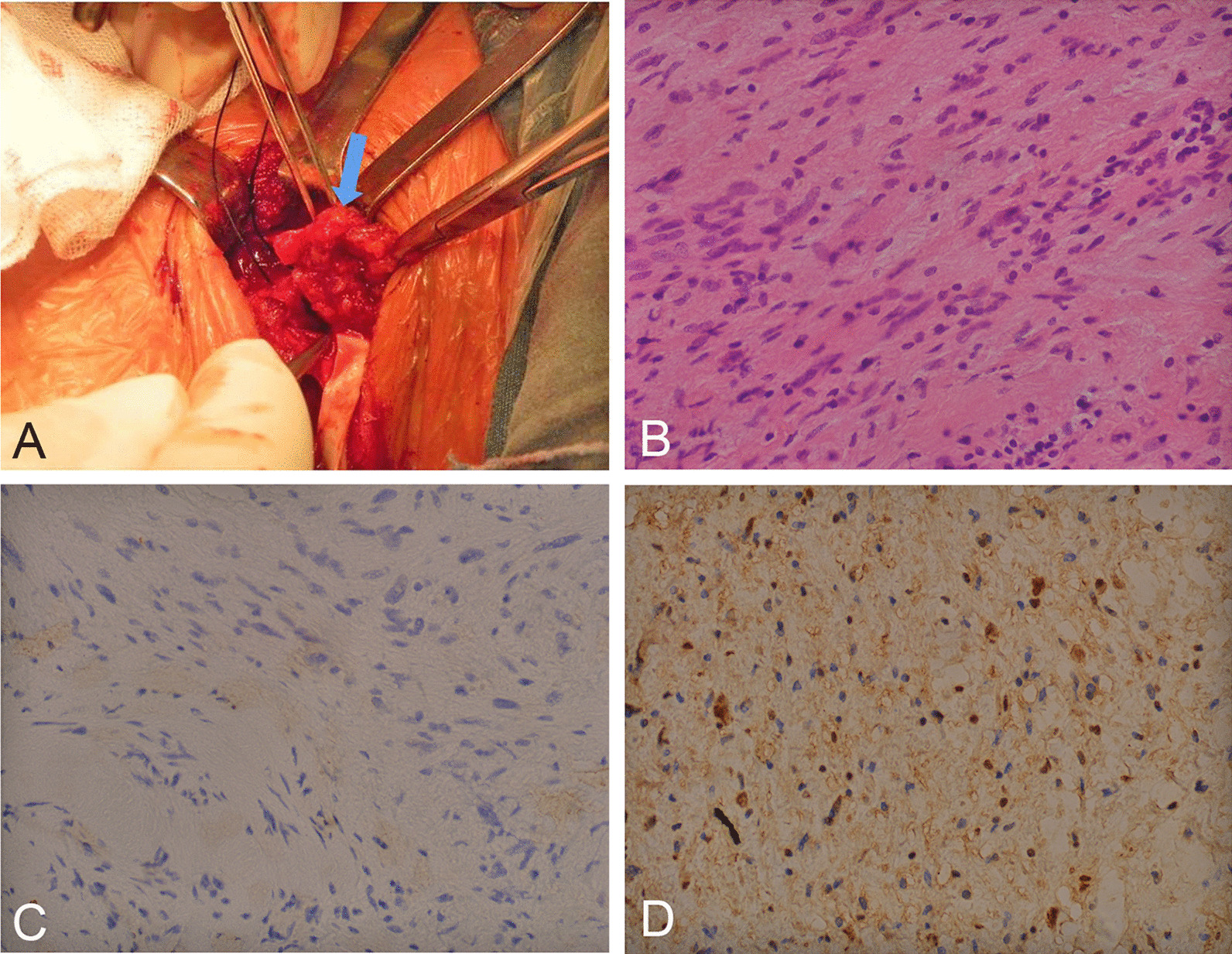
Intraoperative view and pathology of schwannoma. **A** Intraoperative image shows the schwannoma in the C4 CP. The blue arrow indicates the neural lesion. **B** HE (× 400) staining confirmed schwannoma. **C**, **D** NF and S-100 (× 400) staining demonstrating nuclear and cytoplasmic immunoreactivity

MRI (all patients) and CT (4 patients) showed CP masses with enlarged intervertebral foramens (Fig. 2D–F). Pathology confirmed that 72.7% (8/11) of tumours were schwannomas (Fig. 3), 2 were neurofibromas (18.2%), and 1 was a spindle cell tumour (9.1%). Compared with pathology, the diagnostic rates of CP tumours by MRI, CT and high-frequency ultrasound were 72.7% (8/11), 25% (1/4) and 90.9% (10/11), respectively (Table 3).

**Table 3:** CT, MRI and Pathology diagnosis of cervical plexus tumours

Patients	Sex	Age (years)	Mass location (intraoperative)	Pathology	US (n = 11)	CT (n= 4)	MRI (n = 11)
1	Male	50	C4	Schwannoma	CP tumor	Masses located between the internal and external carotid arteries	Neurogenic tumor (C4 level)
2	Female	15	C3	Neurofibromas	CP tumor	–	Neurogenic tumor (C3 level)
3	Female	24	C4	Spindle cell tumour	Lymph nodes enlargement? CP tumor can’t be excluded	–	Lymph nodes enlargement?
4	Female	51	C3	Schwannoma	CP tumor	–	Schwannoma (C3 level)
5	Female	28	C4	Schwannoma	CP tumor	–	Neurogenic tumor (C3 or C4 level)
6	Female	50	C4	Schwannoma	CP tumor	Carotid body tumor	Neurogenic tumor (C4 level)
7	Female	46	C4	Schwannoma	CP tumor	–	Neurogenic tumor (C4 level)
8	Female	54	C4	Schwannoma	CP tumor	Mass behind the Sternocleidomastoid muscle	Chemoreceptor Neoplasia
9	Female	45	C4	Neurofibromas	CP tumor	Mass located in cervical plexus (C3 or C4 level)	Neurogenic tumor (C4 level)
10	Male	51	C3	Schwannoma	CP tumor	–	Schwannoma (C3 or C4 level)
11	Male	34	C4	Schwannoma	CP tumor	–	Lymph nodes enlargement? Neurogenic tumor can’t be excluded
Diagnosis sensitivity (%)					90.90%	25%	72.70%

*US* ultrasound, *CT* computed tomography, *MRI* magnetic resonance imaging

## Discussion

Identification of the CP during ultrasound exam is challenging. According to our experience, two anatomical markers, the C7 vertebra and the bifurcation of the CCA, are very useful for CP identification. The C7 vertebra is unique because it has a posterior tubercle only, which easily distinguishes it from the other cervical vertebrae. The bifurcation of the CCA occurs at the C4 level. After C2–C4 are identified (C1 is usually difficult to identify), the probe can be transversely placed on the neck to show the structure of the cervical nerve root between the anterior and posterior tubercles, and then scanning is performed on the vertical section. Based on the overall scan, if a mass is connected to the thickened cervical nerves (caused by oedema), appearing as a “mouse tail sign” in the long-axis view, it can be considered to originate from the CP.

In our study, we first summarized the high-resolution ultrasound characteristics of CP tumours and found that CP tumours more commonly originate at C4 (72.7%) on the right side (81.8%) and mainly in females (72.7%). Most masses were homogeneously hypoechoic (90.9%) with a clear boundary and envelope integrity (100%). According to previous research, extracranial schwannomas present in the head and neck region account for 25–45% of all benign soft tissue tumours [11]*.* Malignant schwannomas are rare, accounting for only 5% of all soft tissue sarcomas [12]. The pathological results of our research confirmed that all the tumours were benign, with the proportion of schwannomas being 72.7%. Colour Doppler ultrasonography showed that the blood flow signals of 90.9% of patients were grades II and III, demonstrating that benign CP tumours are abundant in blood vessels. Inside the tumours, rich perfusion was observed with contrast-enhanced ultrasound, indicating fast growth of these tumours.

The determination of whether tumours originate from the brachial plexus or CP or from other nerve branches in the neck, as well as the accurate location and diagnosis, is crucial for surgery [13, 14]. Ultrasound plays an important role in the diagnosis of nerve masses. When patients experience cervical pain for unknown reasons, especially when a mass is found or when the limbs are numb, doctors should consider scanning both the CP and brachial plexus to differentiate between injuries, tumours, lymph node hyperplasia and metastasis. When masses are located in the paraspinal space, they are more likely to originate from the CP or brachial plexus rather than lymphoid tissues. Compared with the brachial plexus, the CP is shorter and thinner; therefore, CT and MRI are not satisfactory for demonstrating the CP as the origin of neuromas. For patients with cervical lumps or with pain upon palpation for a long time but without obvious lumps, the possibility of a high-position CP lesion should be considered. In this case, ultrasound can provide valuable information for the clinical strategy.

## Limitation

There are limitations for this study. We included a small sample size of 11 subjects in this study. In addition, some patients did not undergo CT examination, and only 2 cases were done with contrast-enhanced ultrasound. In the future, we will continue to collect the imaging data of CP patients and look forward to providing reference for clinical diagnosis and surgical positioning.

## Conclusion

For CP nerve ultrasonography, the C7 vertebra and bifurcation of the CCA are useful anatomic markers in CP identify. CP tumours were more common in females and generally located at C4 on the right side. For well-trained sonographer, high-frequency ultrasound diagnosis sensitivity of CP neuropathy is accurate and reliable.

## Data Availability

The datasets used and/or analyzed during the current study available from the corresponding author on reasonable request.

## Abbreviations

CP: Cervical plexus
BP: Brachial plexus
MRI: Magnetic resonance imaging
CT: Computed tomography
CCA: Carotid common artery
SD: Standard deviation
